# Effects of Substrate Mechanics on Contractility of Cardiomyocytes Generated from Human Pluripotent Stem Cells

**DOI:** 10.1155/2012/508294

**Published:** 2012-05-09

**Authors:** Laurie B. Hazeltine, Chelsey S. Simmons, Max R. Salick, Xiaojun Lian, Mehmet G. Badur, Wenqing Han, Stephanie M. Delgado, Tetsuro Wakatsuki, Wendy C. Crone, Beth L. Pruitt, Sean P. Palecek

**Affiliations:** ^1^Department of Chemical and Biological Engineering, University of Wisconsin-Madison, 1415 Engineering Drive, Madison, WI 53706, USA; ^2^Department of Mechanical Engineering, Cardiovascular Institute and BioX, Stanford University, 496 Lomita Mall, Stanford, CA 94305, USA; ^3^Department of Engineering Physics, University of Wisconsin-Madison, 1500 Engineering Drive, Madison, WI 53706, USA; ^4^Department of Biology, University of Puerto Rico at Cayey, 205 Avenida Antonio R. Barceló, Cayey, PR 00736, USA; ^5^Department of Physiology and Biotechnology and Bioengineering Center, Medical College of Wisconsin, 8701 Watertown Plank Road, Milwaukee, WI 53226, USA; ^6^InvivoSciences, 510 Charmany Drive, Madison, WI 53719, USA

## Abstract

Human pluripotent stem cell (hPSC-) derived cardiomyocytes have potential applications in drug discovery, toxicity testing, developmental studies, and regenerative medicine. Before these cells can be reliably utilized, characterization of their functionality is required to establish their similarity to native cardiomyocytes. We tracked fluorescent beads embedded in 4.4–99.7 kPa polyacrylamide hydrogels beneath contracting neonatal rat cardiomyocytes and cardiomyocytes generated from hPSCs via growth-factor-induced directed differentiation to measure contractile output in response to changes in substrate mechanics. Contraction stress was determined using traction force microscopy, and morphology was characterized by immunocytochemistry for *α*-actinin and subsequent image analysis. We found that contraction stress of all types of cardiomyocytes increased with substrate stiffness. This effect was not linked to beating rate or morphology. We demonstrated that hPSC-derived cardiomyocyte contractility responded appropriately to isoprenaline and remained stable in culture over a period of 2 months. This study demonstrates that hPSC-derived cardiomyocytes have appropriate functional responses to substrate stiffness and to a pharmaceutical agent, which motivates their use in further applications such as drug evaluation and cardiac therapies.

## 1. Introduction

Cardiovascular disease is the leading cause of mortality in the United States, resulting in 1 of every 2.9 deaths in 2006 [1]. Current mitigation methods are plagued by low effectiveness, and heart transplants are limited by the number of available donor hearts [2, 3]. Engineered heart tissue constructs offer the promise of novel cell-based therapeutic options to restore heart function, but because adult cardiomyocytes have a limited proliferative capacity [4], a source of cardiomyocytes is required for development and implementation of such applications. Human pluripotent stem cells (hPSCs), including human embryonic stem cells (hESCs) [5] and human induced pluripotent stem cells (hiPSCs) [6, 7], offer the potential to produce an unlimited number of cardiomyocytes for tissue engineering and other applications, due to their capacity for unlimited self-renewal and multilineage differentiation. hPSCs have been shown to differentiate to cardiomyocytes, originally via formation of embryoid bodies [8, 9] and more recently, via directed differentiation [10, 11]. These studies utilized molecular techniques to assess cardiomyocyte phenotype, such as examining the temporal expression of the cardiac progenitor markers KDR and PDGFR-*α* [11] or the cardiac transcription factors Nkx2.5 and GATA4 and definitive cardiac markers such as MLC2a, MLC2v, *α*-MHC, MF20, *α*-actinin, cTnT, and cTnI [8, 9]. Immunocytochemistry for the aforementioned definitive markers, which are structural proteins, is also commonly employed to visualize sarcomere definition. The functionality of these cardiomyocytes is typically qualitatively assessed by the presence of spontaneous contraction and can be further characterized using electrophysiology to determine subtype (nodal, atrial, or ventricular) [12].

Response to changes in contractile demand is another critical indicator of functionality. The healthy heart linearly increases its contractile output in response to increased blood flow through a phenomenon known as the Frank-Starling Law [13]. Failure to exhibit this response is a characteristic of mitral stenosis and atrial fibrillation diseased phenotypes [14]. Although communication between neighboring cells undoubtedly plays a role, we hypothesized that singularized cardiomyocytes may maintain this functional response. In order to test this hypothesis, a culture system which can allow for simultaneous modulation of contractile demand and measure of contractile output is required. Polyacrylamide (PA) hydrogels are a well-studied, easily tunable system for studying the effects of substrate stiffness on cell processes [15, 16]. In our case, increasing substrate stiffness is used to increase contractile demand by providing greater resistance to contraction. Addition of fluorescent beads into the prepolymer allows for optical tracking of the displacement of the PA hydrogels. Through computational analysis, bead displacements can be resolved into cell tractions in a technique known as traction force microscopy [17]. This method has been previously used to quantify contractility of quail, rat, and hESC-derived cardiomyocytes [18–21], but the physiological responses of hESC- and hiPSC-derived cardiomyocytes to changes in substrate mechanical properties have not yet been demonstrated. Increasing our understanding of the functional properties of hPSC-derived cardiomyocytes is a critical step to enable their use in further applications, such as *in vitro* drug screening, developmental studies, and engineered heart tissue constructs.

We hypothesized that hPSC-derived cardiomyocytes would increase their contractile output in response to increased substrate stiffness. To test this, we seeded neonatal rat and hPSC-derived cardiomyocytes onto PA hydrogels with a range of stiffnesses and measured their contraction stress using traction force microscopy. We then quantified cell size and shape to identify links between morphology and contraction stress. Finally, we examined changes in hPSC-derived cardiomyocyte contraction stress as a result of drug treatment and increased time in culture.

## 2. Materials and Methods

### 2.1. hPSC Maintenance

Tissue culture polystyrene (TCPS) 6-well plates (Corning) were coated with 0.1% gelatin (Sigma), and irradiated mouse embryonic fibroblasts (MEFs) were seeded at a density of 19,500 cells/cm^2^ in MEF medium. MEF medium consisted of DMEM supplemented with 10% heat-inactivated FBS and 1% MEM nonessential amino acid solution (all components from Life Technologies). hESCs (H9) and hiPSCs (19-9-11) were passaged onto the feeder layers every 5-6 days by exposure to 1 mg/mL collagenase type IV (Life Technologies) in DMEM/F12 (Life Technologies) for 3 minutes at 37°C, followed by mechanical dissociation and centrifugation for 5 minutes at 1000 RPM. hPSCs were maintained in DMEM/F12 culture medium supplemented with 20% KnockOut serum replacer (Life Technologies), 1% MEM nonessential amino acid solution, 1 mM L-glutamine (Life Technologies), 0.1 mM *β*-mercaptoethanol (Sigma), and 4 ng/mL (H9) or 100 ng/mL (19-9-11) human recombinant bFGF (WiCell). At least 1 passage prior to differentiation, hPSCs were seeded onto 8.3 *μ*g/cm^2^ growth factor-reduced Matrigel (BD Biosciences) and maintained in mTeSR1 medium (WiCell). Matrigel coating was performed by resuspending 0.5 mg of Matrigel in 6 mL cold DMEM/F12, adding 1 mL to each well of a 6-well plate and incubating for 1–24 hours at 37°C. hPSCs maintained on Matrigel were passaged every 5-6 days by exposure to Versene (Life Technologies) for 3 minutes at 37°C, followed by mechanical dissociation.

### 2.2. Differentiation of hPSCs to Cardiomyocytes and Characterization via Flow Cytometry

On day −5, hPSCs were dissociated by exposure to Accutase (Life Technologies) for 5 minutes at 37°C to generate single cells. Cells were seeded onto 8.3 *μ*g/cm^2^ Matrigel at a density of 100,000 cells/cm^2^ in mTeSR1 medium supplemented with 5 *μ*M Y-27632 ROCK inhibitor (Stemgent). Cells were maintained in mTeSR1 medium for 2 days. On day −3 through day −1, mTeSR1 was supplemented with 1 *μ*M 6-bromoindirubin-3′-oxime (BIO, Sigma). On day 0, medium was exchanged with RPMI/B27 without insulin (Life Technologies) supplemented with 100 ng/mL Activin A (R&D Systems) and 1% KnockOut serum replacer. 24 hours later, medium was exchanged with RPMI/B27 without insulin supplemented with 5 ng/mL BMP-4 (R&D Systems). On day 5, medium was exchanged with RPMI/B27 without insulin. On day 7 and every 3 days following, medium was exchanged with RPMI/B27 (Life Technologies).

To characterize cardiomyocyte purity via flow cytometry, cells were dissociated into single cells by exposure to 0.25% trypsin-EDTA (Life Technologies) for 5 minutes at 37°C on day 15 of differentiation. Cells were fixed with 1% paraformaldehyde (Electron Microscopy Sciences) in PBS (Life Technologies) for 20 minutes at room temperature. Cells were stained with Troponin T, cardiac isoform Ab-1 mouse monoclonal primary antibody (Thermo Scientific) at 1 : 200 followed by Alexa Fluor 488 goat anti-mouse secondary antibody (Life Technologies) at 1 : 1000 in PBS plus 0.1% Triton X-100 (Sigma) and 0.5% BSA (Sigma). Data were collected on a FACSCalibur flow cytometer (Beckton Dickinson) and analyzed using FlowJo.

1-2 weeks prior to seeding cardiomyocytes onto polyacrylamide hydrogels, cardiomyocytes were dissociated by exposure to 0.25% trypsin-EDTA (Life Technologies) for 5 minutes at 37°C, centrifuged for 3 minutes at 1000 RPM and seeded onto TCPS coated with 0.6 *μ*g/cm^2^ fibronectin (Life Technologies) at a density of 50,000 cells/cm^2^ in EB20 medium. Fibronectin coating was performed by resuspending 36 *μ*g fibronectin in 6 mL PBS, adding 1 mL to each well of a 6-well plate and incubating overnight at 37°C. EB20 medium consisted of DMEM/F12 culture medium supplemented with 20% FBS (Life Technologies), 1% MEM nonessential amino acid solution, 1 mM L-glutamine, and 0.1 mM *β*-mercaptoethanol. The next day and every 3 days following, medium was exchanged with RPMI/B27.

### 2.3. Neonatal Rat Cardiomyocyte Isolation

Based on an approved protocol by the Institutional Animal Care and Use Committee (IACUC) at the Medical College of Wisconsin, cardiomyocytes were isolated from 1-2 day old neonatal Sprague Dawley rats. Ventricular portions of isolated rat hearts were cut into small pieces (~2 mm cubes) and digested with a single (15 min) and repetitive (10 min) incubations with 0.25% trypsin (Life Technologies) and collagenase type IA (75–100 U/mL, Sigma), respectively. We normally obtain ~1 million viable cardiomyocytes from a single heart.

### 2.4. Polyacrylamide Hydrogel Substrate Fabrication

Polyacrylamide (PA) substrates were fabricated using a method adapted from previous studies [15, 22]. Stock solutions of 10% acrylamide (Acros Organics) and 0.03–0.6% bisacrylamide (Fisher) in deionized water were generated and stored at 4°C in amber glass vials. Prior to polymerization, aliquots of each stock solution were brought to room temperature and degassed under vacuum. Polymerization was initiated by 1 : 100 addition of 5% (w/v) ammonium persulfate (APS, Fisher) in deionized water and 5% (v/v) N, N, N′, N′, tetramethylethylenediamine (TEMED, Sigma) in deionized water. 2500 *μ*L of prepolymer was pipetted onto the inverted lid of a glass petri dish (Pyrex) and covered with its base. Both faces of the glass petri dish which contacted the prepolymer were coated with Rain-X (ITW Global Brands), and 1 mm PDMS (Dow Corning) spacers were employed to control gel thickness. After 75 minutes, polymerization was halted by flooding each dish with 50 mM HEPES (Sigma) buffer, pH 8.5. Gels were allowed to swell in HEPES buffer for 1–3 days before continuing.

Circular gels with 1.27 cm diameters were generated from the polymer slabs using a punch cutter (McMaster-Carr). The gels then had two potential fates. For gels designated for traction force microscopy, a 10 *μ*L drop of appropriate prepolymer with the addition of 0.72 *μ*m diameter green fluorescent beads in aqueous solution (Fisher) at 1 : 50 was added to the surface of each gel, and this polymerized under a Rain-X-coated glass coverslip (Fisher) for 75 minutes. Polymerization was halted by addition of 50 mM HEPES buffer, the glass coverslips were removed, and gels were immersed in HEPES buffer for 1–3 days. This two-step polymerization fixed the beads in a single focal plane for improved image quality [22]. Gels designated for immunocytochemistry were immediately immersed in HEPES buffer for 1–3 days and did not undergo the fluorescent bead layer polymerization step to prevent bead bleedthrough into other fluorescent channels from impacting image quality.

To functionalize the gels for protein adhesion, 25 *μ*L of 1 mM N-sulfosuccinimidyl-6-[4′-azido-2′-nitrophenylamino] (Sulfo-SANPAH, Pierce) in HEPES buffer was dried onto all gel surfaces in a 60°C oven for 1.5 hours. Gels were exposed to UV light (OmniCure) at 365 nm, 90 mW/cm^2^ for 2 minutes. The Sulfo-SANPAH addition, drying, and UV exposure steps were repeated once. Gels were transferred to individual wells of 12-well plates, hydrated in PBS, and exposed to germicidal UV light for 20 minutes to sterilize. Gels were coated with 0.6 *μ*g/cm^2^ fibronectin at 37°C overnight. If not used the next day, gels were transferred to 4°C and stored for up to two days.

### 2.5. Polyacrylamide Hydrogel Substrate Characterization

Prepolymer of each bisacrylamide concentration was prepared as described in the previous section, and 400 *μ*L of prepolymer was pipetted to fill a dogbone-shaped Teflon mold. Glass beads with diameters of 30–50 *μ*m (Polysciences, Inc.) were sprinkled over the prepolymer to allow for optical strain measurement during the test, and the prepolymer was covered with a polyethylene terephthalate transparency film. After 75 minutes of polymerization, the mold was disassembled, and samples were stored in HEPES buffer for 1–3 days before mechanical testing to allow the gels to reach hydrostatic equilibrium.

Prior to testing, additional glass beads with diameters of 30–50 *μ*m were adhered to the surface of the samples. The samples were tested in an Instron 5548 MicroTester mechanical testing machine. The samples were secured using self-aligning grips with an abrasive surface at either end to prevent slipping of the sample during testing. The displacement rate of the test was 1 mm/min, which correlated to a strain rate of approximately 0.0025 sec^−1^. This rate was fast enough that evaporation of the surrounding PBS was negligible but slow enough to reduce inertial and viscous effects. A 10 N load cell was used to measure load data at a rate of 1 Hz. The entire system was placed on a pneumatic air table to eliminate noise caused by environmental vibrations.

A temperature-controlled environmental chamber was used during the tests to match *in vivo* conditions as closely as possible. The temperature during the tests was maintained at a constant 37°C via a water jacket surrounding the chamber. The samples were also fully hydrated prior to testing and submerged in PBS during the test to simulate the salinity that the gels would typically experience *in vivo*. Corrections were made to account for the buoyancy of the submerged portions of the testing apparatus. A “buoyancy test” was conducted after each real test to measure the amount of buoyant force that the Instron experienced. This was done by simply removing the sample and running an identical test without any tension between the grips.

Due to the compliant nature of the samples, an optical strain measurement technique was chosen in which the relative displacements of small glass beads embedded within the material or on its surface were measured and used to calculate the strain experienced by the samples. Previous studies have shown that embedding beads within the samples has no effect on the measured modulus of the samples, provided that the beads are small enough, comprise below 1% of the volume, and are evenly distributed within the material [23]. Time-lapse microscopy was used to observe the locations of the beads at designated increments during the tensile test. By measuring the vertical (axial) distance between pairs of beads during these increments, the strains at these times were calculated with Matlab using particle-tracking software developed by Prof. John C. Crocker of the University of Pennsylvania.

The rectangular cross-sections of the samples were measured before and after testing. Before the test, they were measured in multiple places using calipers. After the test, cross-sections were cut from the neck region of the samples and measured optically using a 1.25x microscope objective. Both tests resulted in nearly identical cross-sectional areas, which were then used to calculate stress data. The elastic modulus for each sample is the slope of its stress versus strain curve.

### 2.6. Seeding Cardiomyocytes onto Polyacrylamide Hydrogels

hPSC-derived cardiomyocytes were dissociated by exposure to Accumax (Sigma) for 5 minutes at 37°C, centrifuged for 3 minutes at 1000 RPM, and resuspended in EB20 medium + 1% Antibiotic-Antimycotic (Life Technologies) at a density of 500 cells/*μ*L. Neonatal rat cardiomyocytes were centrifuged for 15 minutes at 650 RPM and resuspended in DMEM + 10% FBS + 1% Antibiotic-Antimycotic at a density of 500 cells/*μ*L. Each hydrogel was washed with PBS and seeded with a 50 *μ*L drop of cell suspension (25,000 cells/gel; seeding density of ~19,700 cells/cm^2^) and placed in a 37°C incubator. 45 minutes later, each well was flooded with 1 mL of appropriate culture medium.

### 2.7. Contraction Stress Measurements and Isoprenaline Treatment

Approximately 24 hours after seeding, contracting cells were imaged to obtain contraction stress data. Imaging was performed within ± 2 days of any timepoint (e.g., “day 30” indicates that measurements were taken between days 28–32 of differentiation). Prior to imaging, each hydrogel was inverted onto a glass-bottomed 35 mm dish (MatTek). Cells were maintained at 37°C during imaging by placement on a heated ring connected to a temperature controller (Fryer). Contracting cells were imaged using a Nikon A1R confocal microscope with a 20x objective. A bidirectional scan speed of 30 frames per second (fps) was used with line averaging of 8 frames to reduce noise, resulting in a capture speed of 3.75 fps. Each cell was imaged for 5 seconds to capture at least 1 contraction cycle.

In some experiments, 9 *μ*M isoprenaline (Sigma) was dosed into the culture medium 5 minutes prior to the start of imaging. As this caused the beating rate to greatly increase, averaging only 4 frames was required to fully capture cell movement, resulting in a capture speed of 7.5 fps. Imaging was completed within 30 minutes of isoprenaline addition.

To quantify contraction stress, one frame corresponding to the maximum point of the contraction cycle, where the cell was at its smallest (the “strain” frame), and one frame corresponding to the minimum point of the contraction cycle, where the cell was at rest (the “null” frame), were identified for each contracting cell. These frames were split into transmitted light and green fluorescent channels and exported using Nikon NIS-Elements software. LIBTRC version 2.4 software, developed by Prof. Micah Dembo of Boston University, was used to determine contraction stress of each cell. The calculations involved have been described in detail elsewhere [17]. Briefly, bead displacements between the “strain” and “null” frames were calculated and loaded into a template file, along with pixel position data for the cell outline in the “strain” frame obtained from ImageJ and numerical values describing the substrate mechanical properties, fluorescent bead characteristics, and image properties. A mesh within the cell outline was created, and the most likely contraction vectors between the “strain” and “null” frames were calculated and converted to contraction stresses. An average of the absolute value of contraction stresses over area generated by each cell at the maximum point of its contraction cycle is reported (avg. contraction stress), as calculated in LIBTRC by the following equation:

(1) $$\text{AVE}\left|T\right|=\frac{1}{A_{\text{cr}1}}\int_{\text{cr}1}\left|T\right|\text{da},$$

where *T* = contraction stress, *A*_cr1_ = total cell area, and a = area of a subsection associated with a particular contraction stress value. When maximum (max) contraction stress is reported, this value is the upper limit of the range of contraction stresses generated by each cell at the maximum point of its contraction cycle. Data sets with <150 bead displacement vectors were rejected as insufficient to accurately determine contraction stress.

### 2.8. Immunocytochemistry and Morphology Characterization

24 hours after seeding, cells were fixed with 16% paraformaldehyde (Electron Microscopy Sciences), diluted to 4% in PBS, for 15 minutes at room temperature. Cells were stored at 4°C in PBS for up to 1 week prior to the initiation of immunocytochemistry. Cells were permeabilized with 0.4% Triton-X 100 (Sigma) in PBS for 1 hour at room temperature and then blocked with 5% nonfat dry milk (Bio-Rad) in PBS for 1 hour at room temperature. Cells were stained with mouse monoclonal anti-*α*-actinin primary antibody (Sigma) at 1 : 500 in 0.4% Triton X-100 in PBS overnight at 4°C. Cells were washed 4 times in PBS for 15 minutes each time. Cells were stained with Alexa Fluor 555 goat anti-mouse secondary antibody (Life Technologies) at 1 : 1000 in 0.4% Triton X-100 in PBS for 1 hour at room temperature. Cells were washed 3 times in PBS for 15 minutes each time, and nuclei were stained with Hoechst 33342 (Life Technologies) at 1 : 5000 in PBS for 5 minutes at room temperature. Prior to imaging, cells were inverted onto a glass coverslip containing one drop of SlowFade Gold antifade reagent (Life Technologies) and sandwiched with another glass coverslip on top. Images were collected on a Nikon A1R confocal microscope with a 20x or 60x water immersion objective.

Images were processed with CellProfiler software using a customized pipeline to obtain cell area and eccentricity. Data were sorted based on cell area, and the top 10% and bottom 10% were excluded to eliminate cell debris and clumps.

### 2.9. Statistical Analysis

Statistical significance was determined using one-way or two-way ANOVA followed by post-hoc Bonferroni tests or linear regression where appropriate. Comparisons with *P* < 0.05 (*), *P* < 0.01 (**), and *P* < 0.001 (***) were determined to be significant. All error bars represent SEM.

## 3. Results

### 3.1. Elastic Moduli of Polyacrylamide Hydrogels Are Physiologically Relevant

**Figure 1:**
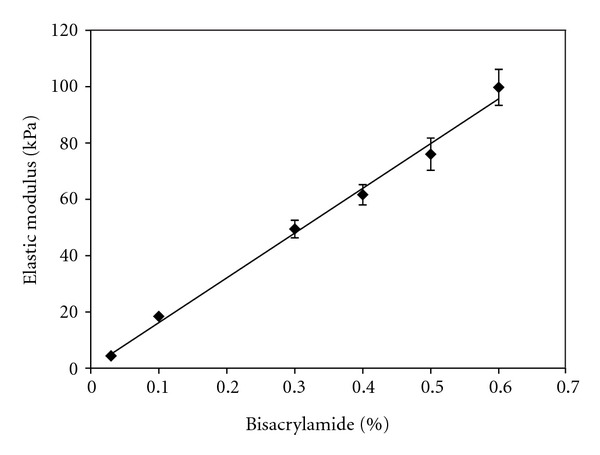
Tensile testing data for polyacrylamide hydrogels shows a linear relationship between elastic modulus and crosslinker concentration. The concentration of acrylamide monomer was held constant at 10% and the concentration of bisacrylamide crosslinker was varied between 0.03–0.6%. Substrate stiffness increased linearly with bisacrylamide concentration over the tested range of compositions. Linear regression demonstrated a significantly nonzero slope (*m* = 159.4 ± 4.6, *P* < 0.0001). *n* = 6–13 for each hydrogel composition and error bars represent SEM.

The elastic modulus, or stiffness, of each polyacrylamide hydrogel composition was determined through tensile testing. The concentration of acrylamide was held constant at 10%, and the concentration of bisacrylamide crosslinker was varied from 0.03–0.6%. Elastic modulus varied linearly with bisacrylamide concentration over this composition range (Figure 1). The moduli of the PA hydrogels used in this study, 4.4 kPa to 99.7 kPa, are physiologically relevant for mimicking the bulk stiffness of cardiac tissue *in vivo*. The elastic moduli of neonatal rat heart tissue were measured to be between 4.0 and 11.4 kPa, and elastic moduli of adult rat heart tissue were found to range from 11.9 to 46.2 kPa [20]. After myocardial infarction, tissue stiffness can increase threefold due to remodeling events and changes in the extracellular matrix composition [24]. Thus, these PA hydrogel substrates encompass a range of moduli representative of developing, mature, and diseased heart tissue. 

### 3.2. Directed Differentiation of hPSCs Yields Pure Populations of Cardiomyocytes

**Figure 2:**
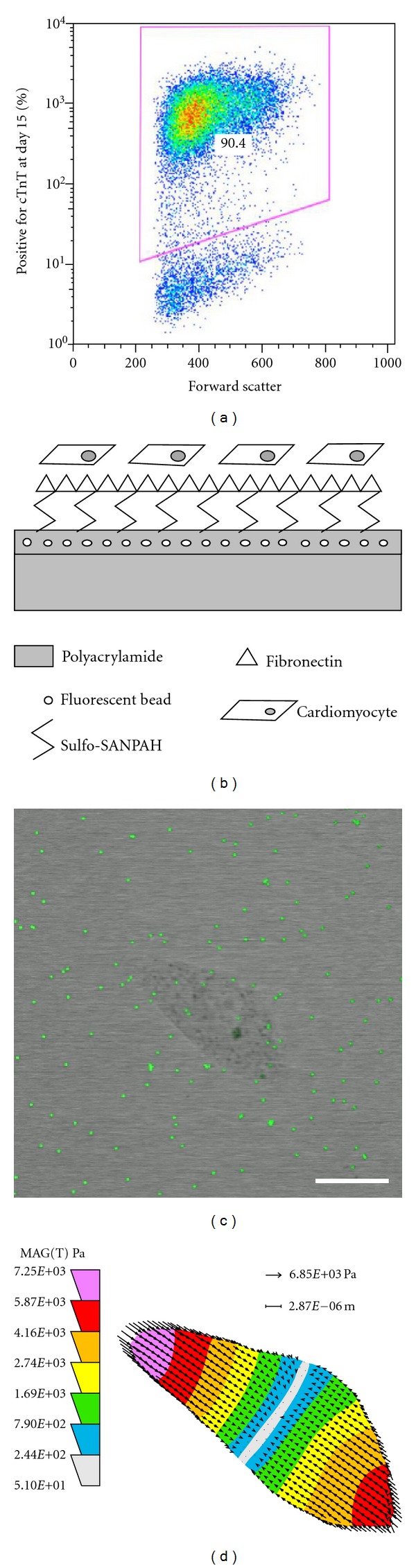
Methods and sample data for obtaining contraction stress measurements. (a) Flow cytometry data shows a typical H9-derived cardiomyocyte population of >90% purity on day 15 of differentiation, demonstrated by the percentage of cells expressing cardiac Troponin T (cTnT). (b) Schematic of polyacrylamide hydrogel cross-section after surface treatment and cell seeding. (c) Merged image shows a contracting D30 19-9-11-derived cardiomyocyte and green fluorescent beads embedded in the 76.0 kPa substrate beneath the cell. The cell was at its maximum point in the contraction cycle. Scale bar = 20 *μ*m. (d) Contraction stress map for the cell pictured in (c) shows the range and localizations of contraction stresses.

To differentiate hPSCs to cardiomyocytes, we implemented a monolayer-based directed differentiation protocol [10, 11] with minor modifications. This protocol cultures undifferentiated cells in medium containing 6-bromoindirubin-3′-oxime (BIO) prior to induction of differentiation by Activin A and BMP-4.  Figure 2(a) demonstrates that H9 hESCs differentiated via this protocol underwent efficient differentiation to cardiomyocytes, with >90% of the population expressing cardiac Troponin T (cTnT) at 15 days postinduction of differentiation. Cells differentiated via this protocol were seeded onto fibronectin-coated PA hydrogels (Figure 2(b)).

### 3.3. Contraction Stress of Cardiomyocytes Increases with Substrate Stiffness

Contracting cells seeded on the hydrogels were imaged with a confocal microscope, permitting simultaneous capture of the transmitted light and green fluorescent channels (Figure 2(c); Supplementary Videos 1–3 (Supplementary materials available on line at doi:10.1155/2012/508294)). Frames corresponding to the maximum and minimum points in the contraction cycle were identified. The transmitted light image corresponding to the maximum contraction point was used to define the contracted cell boundary. The green fluorescent images at the maximum and minimum points were compared using LIBTRC traction force microscopy software, and bead displacements as a result of cell contraction were determined. Along with inputs of numerical values describing the substrate mechanical properties, fluorescent bead characteristics, and image properties (Supplementary Table 1), this information was used to obtain a contraction stress map of each contracting cell (Figure 2(d)). This map shows both the range and localizations of contraction stresses generated by each cell to produce the observed bead movement. When we refer to “maximum (max) contraction stress,” this denotes the upper limit of the range of contraction stresses generated by each cell. In most cases, in order to simplify comparisons between different cells and substrates, an average of the absolute values of contraction stresses over area is reported for each cell, referred to as “average (avg.) contraction stress.” As an example, contraction stresses generated by the cell in Figure 2(d) ranged from 5.10E + 01 to 7.25E + 03 Pa (0.051 to 7.25 mN/mm^2^), and its average contraction stress over area was 2.60E + 03 Pa (2.60 mN/mm^2^).

**Figure 3:**
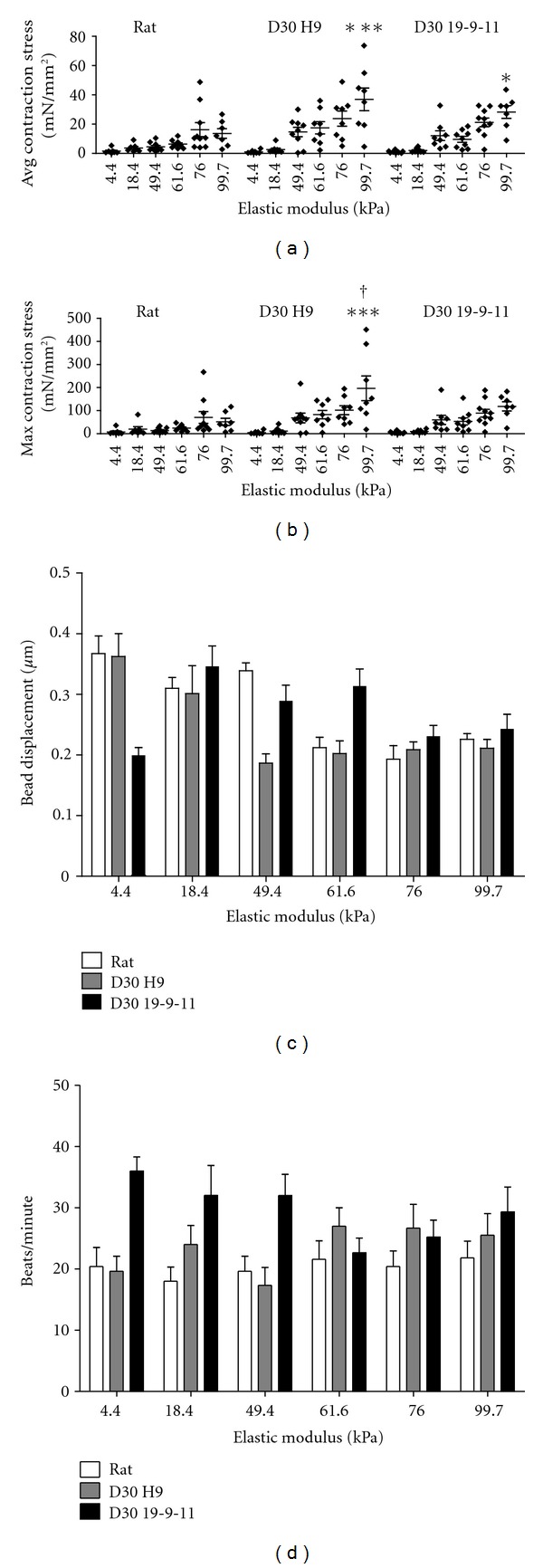
Contraction stress of cardiomyocytes increased with substrate stiffness; bead displacement and beating rate did not increase with stiffness. (a) Average contraction stress of neonatal rat, D30 H9-derived, and D30 19-9-11-derived cardiomyocytes increased with substrate stiffness. Each data point represents an average of the absolute values of contraction stresses over area for a single cell at the maximum point of its contraction cycle. Mean contraction stress values were significantly affected by stiffness via one-way ANOVA (overall *P* = 0.0018 for rat and overall *P* < 0.0001 for D30 H9 and D30 19-9-11). ***(*P* < 0.001) and *(*P* < 0.05) indicate statistically significant differences relative to neonatal rat cardiomyocytes on the same stiffness. (b) Maximum contraction stress of neonatal rat, D30 H9-derived, and D30 19-9-11-derived cardiomyocytes increased with substrate stiffness. Each data point represents the upper limit of the range of contraction stresses generated by a single cell at the maximum point of its contraction cycle. ***(*P* < 0.0001) and ^†^(*P* < 0.05) indicate statistically significant differences relative to neonatal rat and D30 19-9-11-derived cardiomyocytes, respectively, on the same stiffness. (c) Displacement of fluorescent beads occurred to a similar extent on all stiffnesses. Each data point represents an average of the absolute values of bead displacement for a single cell, including both near- and far-field beads. For all cell lines, substrate stiffness significantly affected bead displacement using one-way ANOVA (overall *P* < 0.0001, *P* = 0.0001, *P* = 0.0003 for rat, D30 H9, and D30 19-9-11 resp.), with an overall significant decreasing (rat and D30 H9) or nonsignificant increasing (D30 19-9-11) trend via linear regression. (d) Beating rate was similar on all stiffnesses. Substrate stiffness did not significantly affect beating rate via one-way ANOVA for each cell line (overall *P* = 0.92, *P* = 0.18, *P* = 0.07 for rat, D30 H9, and D30 19-9-11, resp.). For (a)–(d), *n* = 7–11 cells for each stiffness and error bars represent SEM.

To obtain a baseline for cardiomyocyte contractility on our PA hydrogels, we first measured contraction stresses of individual neonatal rat cardiomyocytes. The average contraction stress of these cells increased with substrate stiffness, and means for the stiffnesses exhibited statistically significant differences using one-way ANOVA (overall *P* = 0.0018) (Figure 3(a)). A similar stiffness-dependant profile was observed when comparing maximum contraction stress of neonatal rat cardiomyocytes (Figure 3(b)). Next, we measured the contraction stresses of D30 (30 days postdifferentiation) H9 hESC-derived cardiomyocytes and D30 19-9-11 hiPSC-derived cardiomyocytes. In both types of human pluripotent stem cell-derived cardiomyocytes, average contraction stress increased with substrate stiffness, and means had statistically significant differences using one-way ANOVA (H9 overall *P* < 0.0001 and 19-9-11 overall *P* < 0.0001) (Figure 3(a)). D30 H9- and D30 19-9-11-derived cardiomyocytes generated statistically significantly higher magnitudes of average contraction stress than rat cardiomyocytes on the 99.7 kPa stiffness (*P* < 0.001 for D30 H9 versus rat and *P* < 0.05 for D30 19-9-11 versus rat). We also compared the maximum contraction stresses generated by D30 H9- and D30 19-9-11-derived cardiomyocytes. Stiffness-dependant profiles were similar to those of average contraction stress, and statistically significant differences between cell lines were observed only on the 99.7 kPa gel (*P* < 0.001 for D30 H9 versus rat and *P* < 0.05 for D30 H9 versus D30 19-9-11) (Figure 3(b)).

Contraction stress also increased with substrate stiffness in 19-9-11-derived cardiomyocytes subject to a modification in the derivation protocol, in which BIO was omitted from the culture medium and Matrigel was added on days −3 and 0. On the two stiffest substrates, the Matrigel protocol resulted in significantly higher levels of average contraction stress relative to the BIO protocol (Supplementary Figure 1(A)). To examine the effect of PA hydrogel culture time, we measured the contraction stresses of neonatal rat cardiomyocytes at 1 day and 3 days after seeding. Average contraction stress increased with stiffness at both time points and was not significantly different on any stiffness at 3 days relative to 1 day (Supplementary Figure 1(B)).

To test whether cells in clusters act cooperatively to generate more contraction stress than single cells, we plated incompletely dissociated H9-derived cardiomyocytes on PA hydrogels, resulting in a mixture of single cells and clumps containing approximately 2–20 cells. We found that cell clumps contracted in a coordinated manner (Supplementary Video 4), suggesting electrical coupling but did not produce more average contraction stress than single cells (*P* = 0.10) (Supplementary Figure 2).

### 3.4. Bead Displacement and Beating Rate do Not Increase with Substrate Stiffness

Next we examined the extent to which the cardiomyocytes displaced fluorescent beads embedded in the PA hydrogels, as this is a critical input for determining contraction stress. Bead displacement was detected on all stiffnesses used in this study, with average displacement magnitudes per cell ranging from 0.2–0.35 *μ*m (Figure 3(c)). These average magnitudes per cell represent both near-field beads, which were within or close to the cell boundary and were displaced greater distances than the average upon contraction and far-field beads, which were displaced only slight distances upon contraction. For all cell lines, substrate stiffness significantly affected bead displacement using one-way ANOVA (overall *P* < 0.0001, *P* = 0.0001, *P* = 0.0003 for rat, D30 H9, and D30 19-9-11, resp.); however, linear regression revealed a statistically significant decreasing trend in bead displacement with stiffness for rat and D30 H9 and a nonstatistically significant increasing trend for D30 19-9-11.

We then calculated the beating rate for contracting cells on all stiffnesses, and we found that substrate stiffness did not significantly affect beating rate (Figure 3(d)). Mean beating rates of rat, D30 H9-, and D30 19-9-11-derived cardiomyocytes on all stiffnesses were not significantly different using one-way ANOVA for each cell line (overall *P* = 0.92, *P* = 0.18, *P* = 0.07 for rat, D30 H9, and D30 19-9-11, resp.).

### 3.5. Substrate Stiffness Affects Cardiomyocyte Area

**Figure 4:**
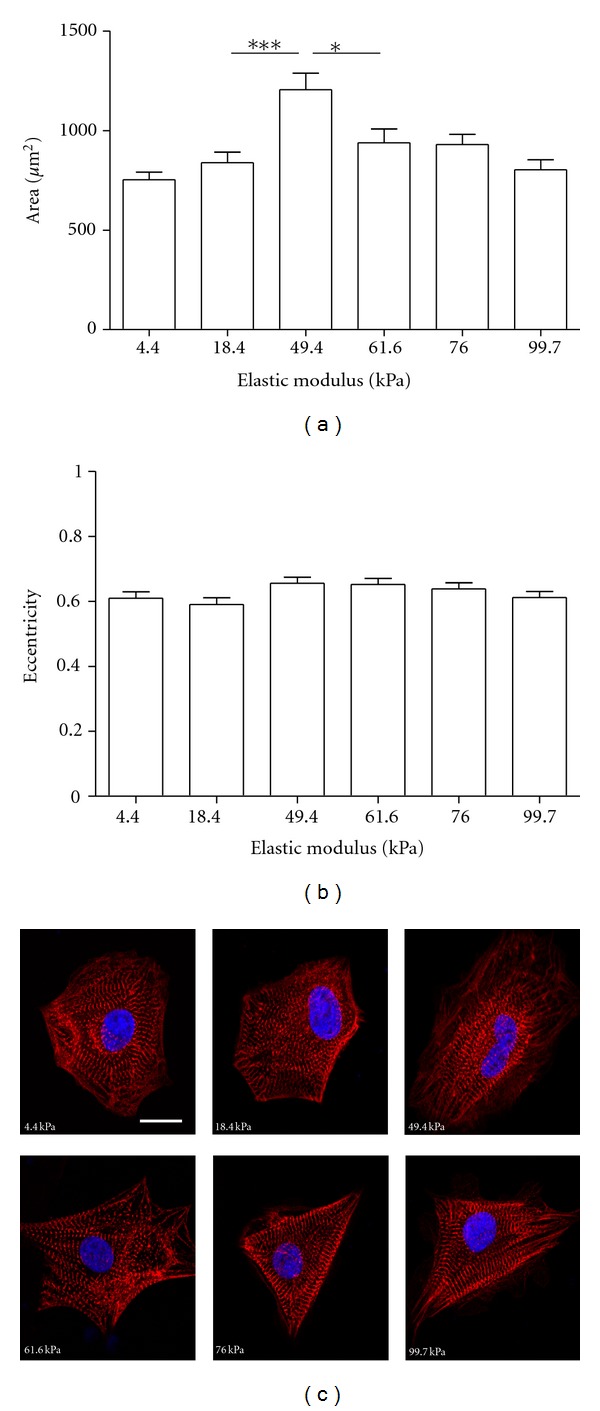
Morphological characterization of D30 19-9-11-derived cardiomyocytes on polyacrylamide hydrogels. Cells were seeded onto the hydrogels at 1 month postdifferentiation, fixed 24 hours later, and immunostained for *α*-actinin. Morphology was characterized using CellProfiler software. (a) Substrate stiffness significantly affected cell area (overall *P* < 0.0001 via one-way ANOVA). Cell area peaked on the 49.4 kPa hydrogel. ***(*P* < 0.001) and *(*P* < 0.05) indicate statistically significant differences. (b) Substrate stiffness did not significantly affect eccentricity (overall *P* = 0.12 via one-way ANOVA). For (a) and (b), *n* = 81–112 cells per stiffness and error bars represent SEM. (c) Representative images show sarcomere organization on all stiffnesses. *α*-actinin is shown in red, and nuclei (stained with Hoechst) are shown in blue. Scale bar = 10 *μ*m.

We next examined the morphology of neonatal rat and hPSC-derived cardiomyocytes on all stiffnesses in an effort to identify correlations between contraction stress and morphology. We used immunocytochemistry to stain cells for *α*-actinin, a protein which localizes at the Z-band of sarcomeres. We imaged cells at low magnification (20x) to capture many cells in a frame and quantified their morphology using CellProfiler software and at high magnification (60x) to visualize the sarcomere structure of individual cells. We quantified cell area and eccentricity or degree of elongation (0 = circular, 1 = fully elongated), to represent cell size and shape. For D30 19-9-11-derived cardiomyocytes, substrate stiffness significantly affected mean cell area (overall *P* < 0.0001 via one-way ANOVA). Cell area was greatest on the 49.4 kPa PA hydrogel, and this represents a statistically significant difference from areas on the 18.4 and 61.6 kPa hydrogels (Figure 4(a)). Cell eccentricity averaged ~0.6 on all substrate stiffnesses and did not differ significantly with stiffness (overall *P* = 0.12 via one-way ANOVA) (Figure 4(b)). Well-defined sarcomeres were observed on all substrate stiffnesses (Figure 4(c)). Similar observations for morphology and sarcomere organization were made using neonatal rat, D30 H9-derived, and D60 H9-derived cardiomyocytes (Supplementary Figures 3, 4, and 5), with key differences being a higher eccentricity value (~0.8) for neonatal rat cardiomyocytes on all stiffnesses and an emerging dependence of eccentricity on stiffness for D60 H9-derived cardiomyocytes.

### 3.6. hPSC-Derived Cardiomyocytes Respond Appropriately to Isoprenaline

**Figure 5:**
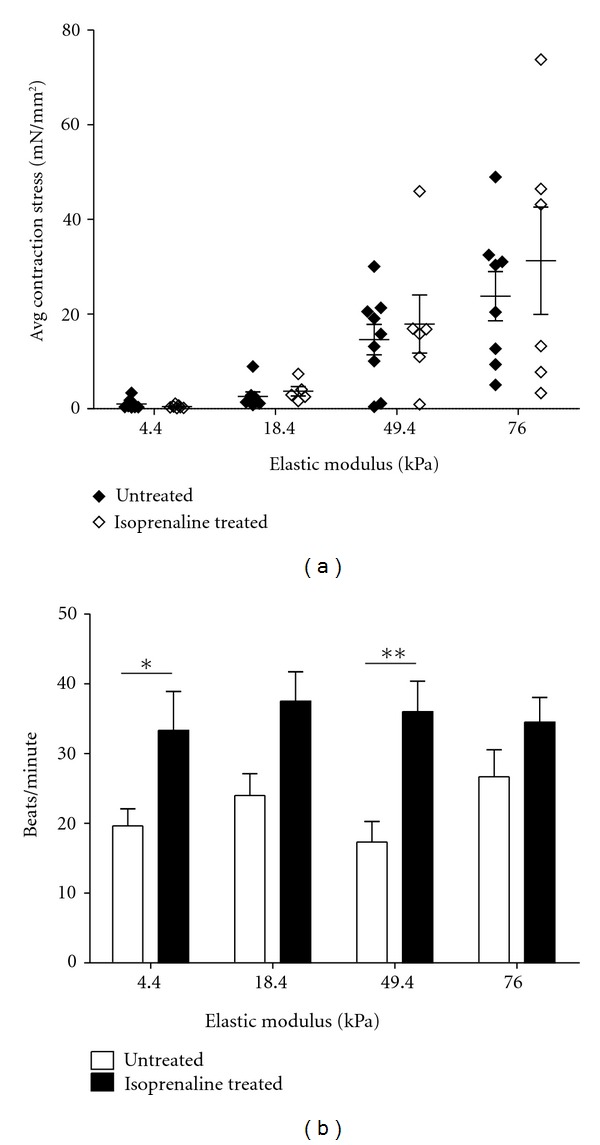
Isoprenaline treatment increased beating rate but not contraction stress of D30 H9-derived cardiomyocytes. Cells were seeded onto polyacrylamide hydrogels at 1 month postdifferentiation and imaged 24 hours later to obtain contraction stress and beating rate. One population was untreated, and the other was treated with 9 *μ*M isoprenaline for 5 minutes prior to imaging. (a) Average contraction stress was not significantly affected by isoprenaline treatment (overall *P* = 0.40 for effect of treatment via two-way ANOVA). *n* = 8-9 cells per stiffness for untreated and 5–7 cells per stiffness for isoprenaline treated. (b) Beating rate significantly increased upon isoprenaline treatment (overall *P* < 0.0001 for effect of treatment via two-way ANOVA). **(*P* < 0.01) and *(*P* < 0.05) indicate statistically significant differences. *n* = 9–11 cells per stiffness for untreated and 6–9 cells per stiffness for isoprenaline treated. For (a) and (b), error bars represent SEM.

Another key indicator of cardiomyocyte functionality is that contractility should change appropriately in response to pharmaceutical agents. Isoprenaline, a *β*_1_-adrenoceptor agonist, has been demonstrated to increase both beating rate and force of cardiomyocytes [25, 26]. We dosed 9 *μ*M isoprenaline into the culture medium of hPSC-derived cardiomyocytes on PA hydrogels prior to imaging. Upon isoprenaline treatment, the mean value of average contraction stress did not change significantly on any stiffness (overall *P* = 0.40 for effect of treatment via two-way ANOVA), although it was slightly higher than that of untreated cells on the 18.4, 49.4, and 76.0 kPa substrates (Figure 5(a)). The beating rate was higher on all substrates and was significantly affected by isoprenaline treatment (overall *P* < 0.0001 for effect of treatment via two-way ANOVA), with a statistically significant increase on the 4.4 and 49.4 kPa substrates in the treated versus untreated populations (Figure 5(b)). These results were verified with neonatal rat cardiomyocytes, with a nonstatistically significant impact of isoprenaline treatment on contraction stress and a significant impact on beating rate on the 4.4, 76.0, and 99.7 kPa substrates (Supplementary Figure 6).

### 3.7. Contraction Stress of hPSC-Derived Cardiomyocytes Remains Stable with Time

**Figure 6:**
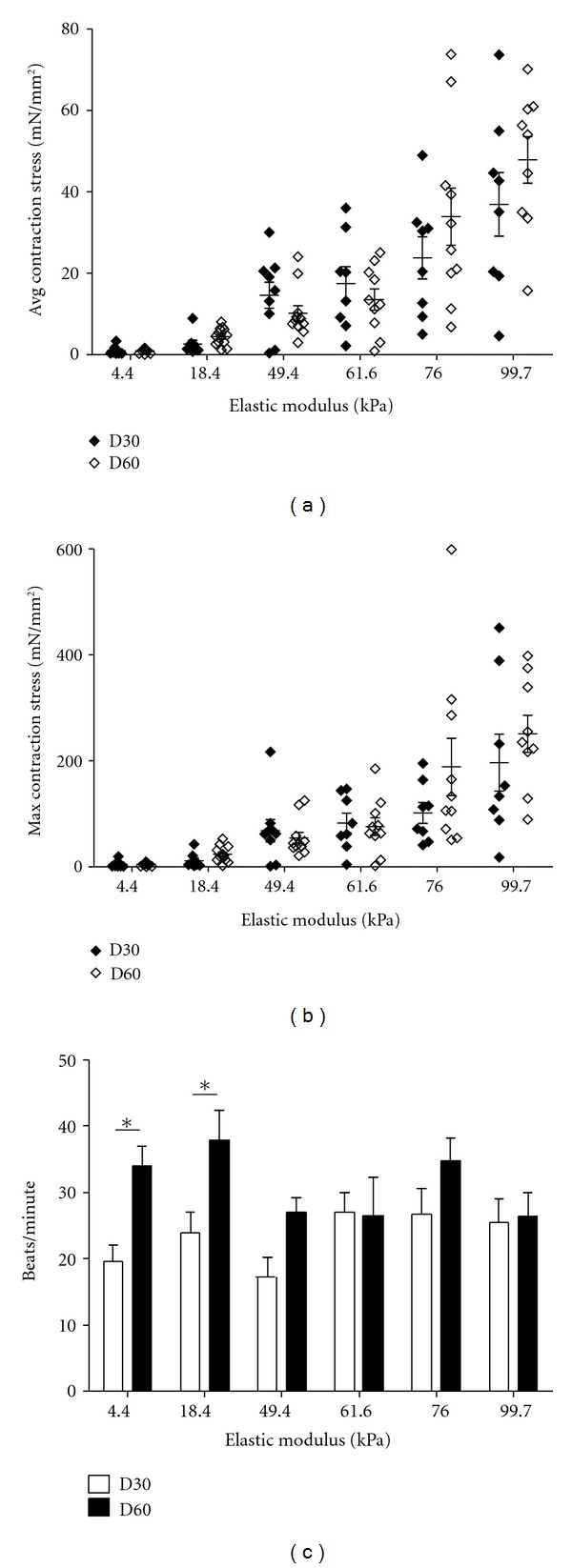
Contraction stress remained stable with time past differentiation, while beating rate changed on soft substrates. H9-derived cardiomyocytes were seeded onto polyacrylamide hydrogels at 30 and 60 days postdifferentiation and imaged 24 hours later to obtain contraction stress and beating rate. (a) Average contraction stress increased with substrate stiffness but was not significantly different on any stiffness at D30 and D60 (overall *P* = 0.29 for effect of time via two-way ANOVA). (b) Maximum contraction stress increased with substrate stiffness but was not significantly different on any stiffness at D30 and D60 (overall *P* = 0.14 for effect of time via two-way ANOVA). For (a) and (b), *n* = 8-9 cells per stiffness for D30 and 9–13 cells per stiffness for D60. (c) Stiffness did not significantly affect beating rates of D60 H9-derived cardiomyocytes (overall *P* = 0.13 via one-way ANOVA), but beating rate increased on soft substrates between D30 and D60. *(*P* < 0.05) indicates statistically significant differences. *n* = 8–11 cells per stiffness for D30 and 10–13 cells per stiffness for D60. For (a)–(c), error bars represent SEM.

It has been demonstrated that hPSC-derived cardiomyocytes increase their functional maturity with time through the metrics of ion channel expression and electrophysiology [27], but effects of culture time on contraction stress generation are unknown. To investigate this, we measured contraction stresses of H9-derived cardiomyocytes at both day 30 (D30) and day 60 (D60) of differentiation. Time in culture was not found to significantly affect average or maximum contraction stress at these two time points via two-way ANOVA (overall *P* = 0.29 and overall *P* = 0.14 for avg. and max, resp.) (Figures 6(a) and 6(b)). Similar observations were made when comparing average and maximum contraction stresses of D30 and D60 19-9-11-derived cardiomyocytes (Supplementary Figures 7(A) and 7(B)), with no significant difference observed over time on any stiffness (overall *P* = 0.20 (avg.) and overall *P* = 0.97 (max) for effect of time via two-way ANOVA). Stiffness did not significantly affect beating rates of D60 H9-derived cardiomyocytes (overall *P* = 0.13), but a statistically significant increase in beating rate occurred on the 4.4 and 18.4 kPa substrates between D30 and D60 (Figure 6(c)). A similar observation was made in D60 19-9-11-derived cardiomyocytes, with a nonsignificant impact of stiffness on beating rate at D60; however, statistically significant decreases in beating rate were detected on the 18.4 and 49.4 kPa substrates between D30 and D60 (Supplementary Figure 7(C)).

## 4. Discussion

These results demonstrate that contraction stress of cardiomyocytes generated from hPSCs increases with substrate stiffness. We further show that beating rate and morphology are not linked to this functional response. Finally, we demonstrate that hPSC-derived cardiomyocytes respond to isoprenaline, and that contraction stress remains stable during maintenance in culture. By demonstrating similarities in the functional responses of native and hPSC-derived cardiomyocytes to substrate stiffness and isoprenaline treatment, we hope to motivate the use of hPSC-derived cardiomyocytes in further applications such as drug discovery and regenerative medicine.

The increase of contraction stress with substrate stiffness is predicted by the Frank-Starling Law, which states that the heart will increase its contractile output in response to increased demand [13]. Although this law pertains to the intact organ, we demonstrated that singularized cardiomyocytes also exhibit this functional response by increasing their contraction stress on substrates where increased stiffness provides greater resistance to contraction. The effect of contraction stress increasing with substrate stiffness has been previously demonstrated in rat cardiac tissue monolayers [20] and digested chicken heart tissue [28]. In contrast, other studies demonstrated that contraction stress of neonatal rat cardiomyocytes [19] and contractile work of quail cardiomyocytes [18] peaked on 10 kPa substrates, but differences in cell source, extracellular matrix, measurement timing, and/or electrical stimulation existed between these studies and ours and could account for changes in contractile behavior. Importantly, the magnitudes we measured for average contraction stress at maximum contractile cycle points are in agreement with previous studies of primary human and rat cardiomyocytes. Hasenfuss et al. reported a peak twitch tension for human myocardium of 44 ± 11.7 mN/mm^2^, and similarly 56.4 ±  4.4 mN/mm^2^ for rat myocardium [29]. Lin et al. measured a maximal contraction stress of adult rat cardiomyocytes of 23.7 ± 8.6 mN/mm^2^ [30]. We demonstrated that average contraction stress increases with stiffness both at 1 day and 3 days after seeding onto PA hydrogels in neonatal rat cardiomyocytes, but long-term contractile properties of individualized cells are not known. Others have demonstrated that the percentage of beating cardiomyocytes *in vitro* is inversely proportional to substrate stiffness and declines with time on stiff substrates [18, 19, 31]. *In vivo*, fibrosis of heart tissue after myocardial infarction is a contributing factor to heart failure [32]. These are indications that cardiomyocytes cultured for extended times on stiff substrates may have difficulty maintaining a healthy contractile phenotype.

Comparison of average contraction stresses of single cells and small clumps of 2–20 cells failed to reveal a relationship between contraction stress and clump size. The cells in our clumps were randomly oriented, often with multiple axes of contraction. Topographic control, such as nanogrooved surfaces, may encourage alignment and anisotropic contraction of cardiomyocyte aggregates [33].

Cardiomyocytes generated similar extents of bead displacement on all substrates, suggesting that cells are able to generate similar levels of strain across the range of substrate stiffnesses evaluated in this study. A contributing factor to observed variations in bead displacement could be differences in bead distribution across the surfaces, which results in different ratios of near-field versus far-field beads in each data set. To better control marker position, one could micropattern dots onto hydrogels instead of embedding beads, similar to the approach used by Balaban et al. [34].

In neonatal rat, D30 H9- and D30 19-9-11-derived cardiomyocytes, substrate stiffness did not significantly affect beating rate. For all cell types and stiffnesses, the average beating rate varied between about 20–35 beats/minute. This is much slower than the average human embryonic heart rate of 80–196 beats/minute [35], the average resting adult heart rate of 60–100 beats/minute, and reported beating rates of 50–70 beats/minute for hPSC-derived embryoid bodies [9]. Electrical pacing of hPSC-derived cardiomyocytes may be used to obtain physiologically-relevant beating rates [12].

We found that, for cardiomyocytes differentiated from all hPSC lines, spread area peaked at an intermediate stiffness (either 49.4 kPa or 61.6 kPa). An increase in cell area with stiffness up to 34 kPa was observed in a previous study in embryonic quail cardiomyocytes cultured on PA hydrogels for 4 or 24 hours [18], which is consistent with our observations. In another study with neonatal rat cardiomyocytes on PA hydrogels of 1–50 kPa, stiffness did not affect the area of cells fixed 7 days after seeding [19].

There are several common descriptors of cardiomyocyte shape, including eccentricity, circularity index, and aspect ratio, all of which serve to describe elongation of the cells. *In vivo*, neonatal rat cardiomyocytes have an elongated shape and an aspect (length-to-width) ratio of 7 : 1, translating to an eccentricity value approaching 1. *In vitro*, however, cardiomyocytes undergo a shape change as they adapt to 2D culture and their eccentricity lowers [36, 37]. We did not observe a change in eccentricity with substrate stiffness in rat, D30 H9- and D30 19-9-11-derived cardiomyocytes, thus providing another indication that all substrates used in this study provide an environment similarly conducive for beating. In D60 H9-derived cardiomyocytes, eccentricity exhibited a statistically significant increase with stiffness, but this shape change did not manifest in greater contraction stress generation compared to D30. Neonatal rat cardiomyocytes exhibited an eccentricity of about 0.8 on all stiffnesses, while hPSC-derived cardiomyocytes possessed an eccentricity of about 0.6. This is consistent with the expectation that cells cultivated *in vivo* should have a higher eccentricity value than those cultivated *in vitro*.

Well-defined sarcomeres were observed on all stiffnesses, suggesting that degree of sarcomere organization was not linked to contraction stress production. The representative images demonstrate that, although we quantified general trends in cell size and shape, cell morphology was very heterogeneous across the population. Additionally, most of the hPSC-derived cardiomyocytes possessed multiple myofibril axes. Although cardiomyocyte size and shape do not appear to directly impact contraction stress on the substrates evaluated in this study, they may contribute to the large variability in our contraction stress measurements. Others have demonstrated that cell shape impacts traction stress distribution and magnitudes in NIH 3T3 cells and smooth muscle cells [38, 39]. To control cardiomyocyte size and shape, Bray et al. used microcontact printing of 2500 *μ*m^2^ fibronectin islands and demonstrated that cells on the native aspect ratio of 7 : 1 exhibit directional anisotropy [37]. Adapting a similar fibronectin micropatterning approach may reduce variability in morphology of hPSC-derived cardiomyocytes and by extension decrease variability in contraction stress.

A potential application of hPSC-derived cardiomyocytes is identification of drugs which are toxic to cardiomyocytes or impact their contractility. We treated hPSC-derived cardiomyocytes with isoprenaline to demonstrate that these cells respond appropriately to this drug by increasing their beating rate. Significant effects of isoprenaline treatment on average contraction stress were not observed in this study. We measured contraction stress values from two separate populations, one untreated and one isoprenaline treated. Considering the heterogeneity of our contraction stress measurements, it may be necessary to take untreated and treated measurements from the same population to accurately quantify the contractile response of a single cell. This could be accomplished using a perfusion system, where drugs could be introduced into the medium during measurement without disturbing the position of the substrate and attached cells.

Others have demonstrated that hESC-derived cardiomyocytes mature over time periods up to 110 days in culture, as evidenced by their ultrastructure and withdrawal from the cell cycle [40] as well as their ion channel expression and electrophysiology [27]; however, these cells do not reach a maturation state characteristic of adult cardiomyocytes. We measured contraction stress of hPSC-derived cardiomyocytes at 1 and 2 months postdifferentiation (D30 and D60), likely corresponding to an intermediate stage of maturation and discovered that average and maximum contraction stresses remained stable over this period. Analyses of hPSC-derived cardiomyocyte shape and beating rate at D30 and D60, however, revealed that some properties of these cells are dynamic with respect to time. As new metrics are discovered for measuring hPSC-derived cardiomyocyte maturation, we hope to link contraction stress generation to structural or functional changes which occur over time. Understanding the development of functional responses of these cells will be critical if we hope to use them as a model system for adult cardiomyocyte behavior.

In summary, this study demonstrates that hPSC-derived cardiomyocytes on stiff substrates generate more contraction stress than those on soft substrates. Beating rate and morphology were not directly linked to substrate-dependent effects on contractility. When treated with isoprenaline, hPSC-derived cardiomyocytes exhibited increased beating rate. The baseline contraction stress of these cells remained stable for up to 2 months in culture. Considering their appropriate contractile responses, hPSC-derived cardiomyocytes have the potential to be an integral component of engineered heart tissue constructs or *in vitro* drug studies, among other applications.

## Supplementary Material

The Supplementary Material for this Research Article contains videos of beating cardiomyocytes on polyacrylamide hydrogels (Supplementary Videos 1-4), additional contractility data (Supplementary Figures 1-2 and 6-7), additional morphology characterization data (Supplementary Figures 3-5), and a table of parameters used as inputs to determine contraction stress (Supplementary Table 1).
