# Supplementary figures and images for: Effects of Substrate Mechanics on Contractility of Cardiomyocytes Generated from Human Pluripotent Stem Cells
Source: Int J Cell Biol. 2012 May 9;2012:508294. doi: 10.1155/2012/508294 (PMC3357596; doi:10.1155/2012/508294)

**A**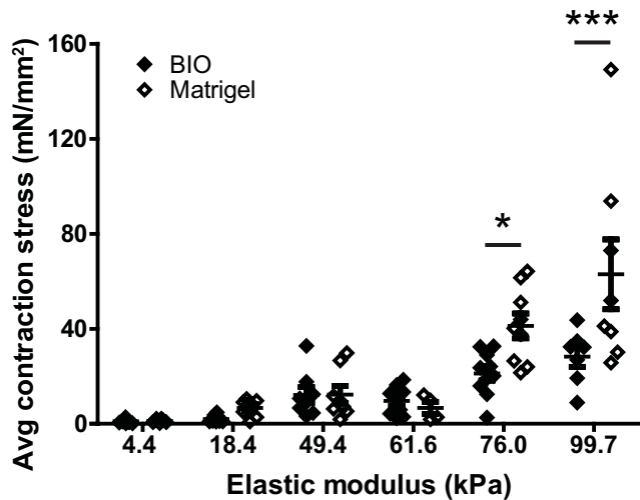**B**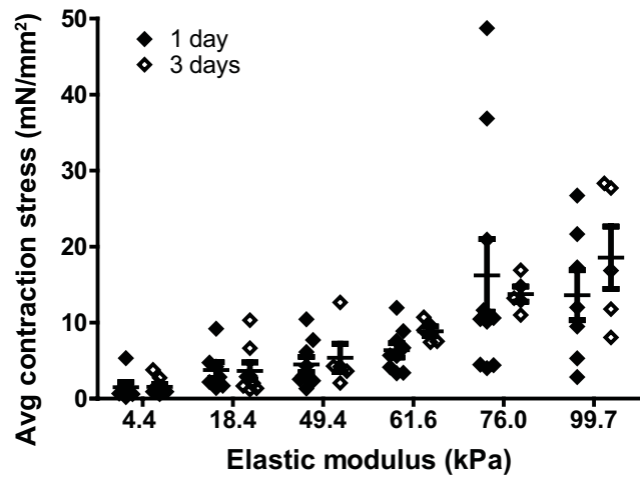

Supplement: Supplementary file 1 — The Supplementary Material for this Research Article contains videos of beating cardiomyocytes on polyacrylamide hydrogels (Supplementary Videos 1-4), additional contractility data (Supplementary Figures 1-2 and 6-7), additional morphology characterization data (Supplementary Figures 3-5), and a table of parameters used as inputs to determine contraction stress (Supplementary Table 1). [file 508294.f1.pdf]

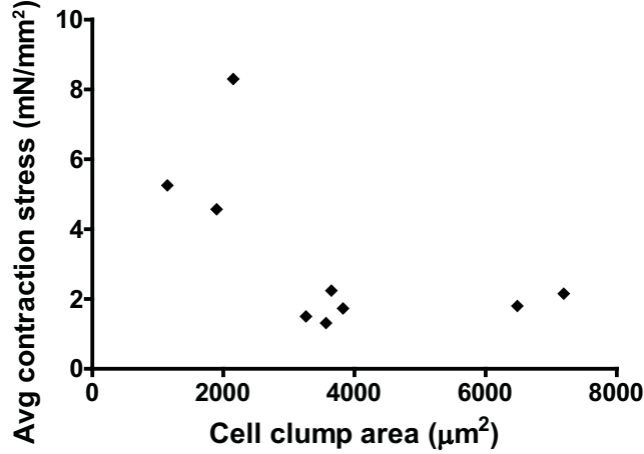

Supplement: Supplementary file 2 [file 508294.f2.pdf]

A

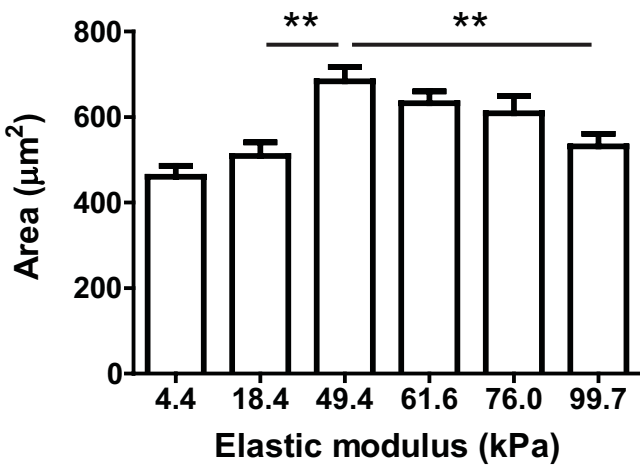

B

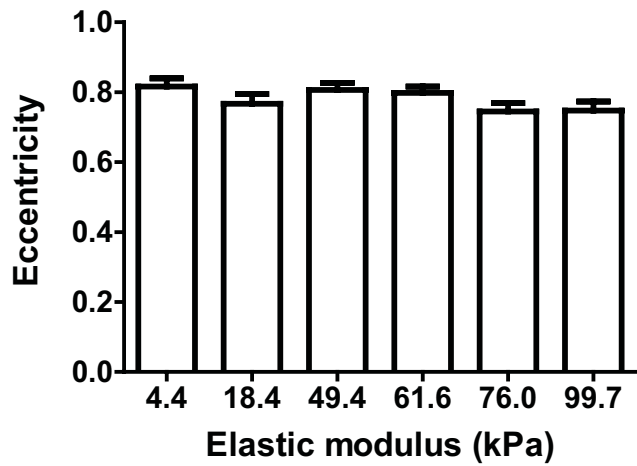

C

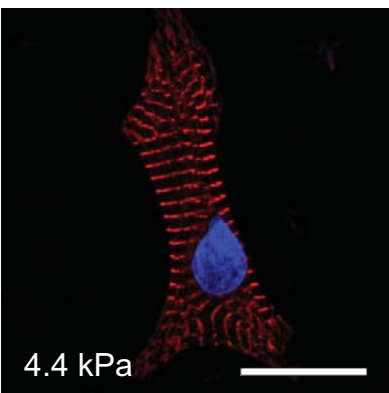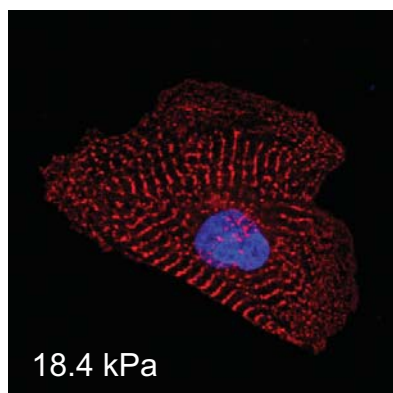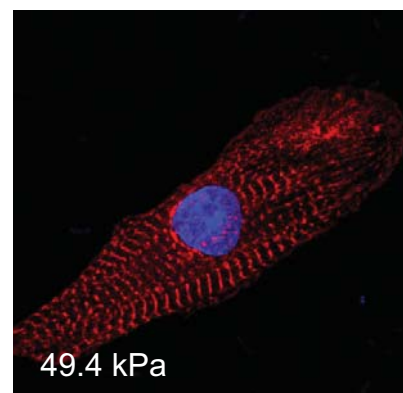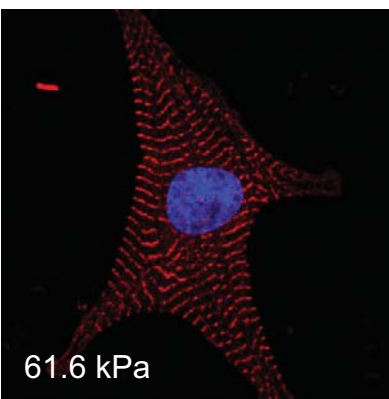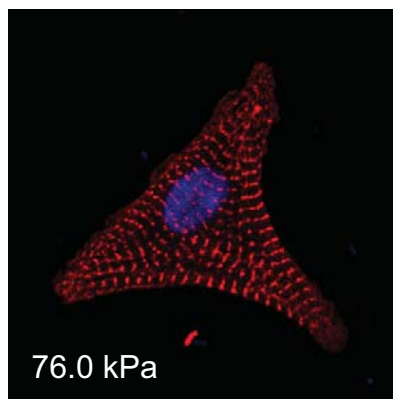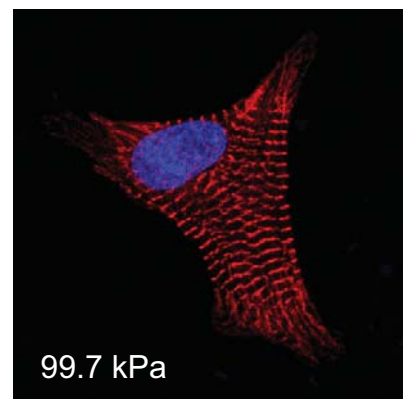

Supplement: Supplementary file 3 [file 508294.f3.pdf]

A

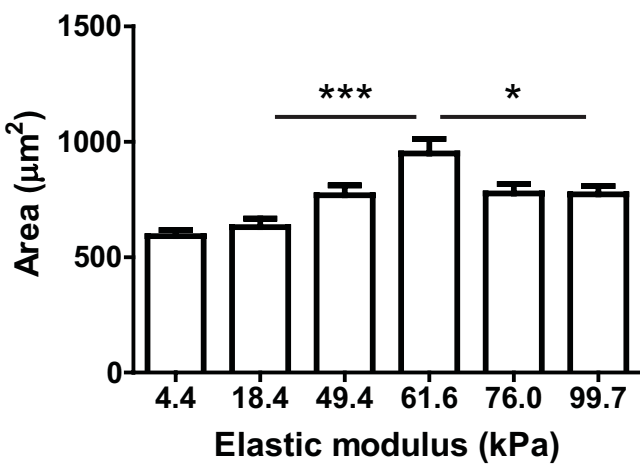

B

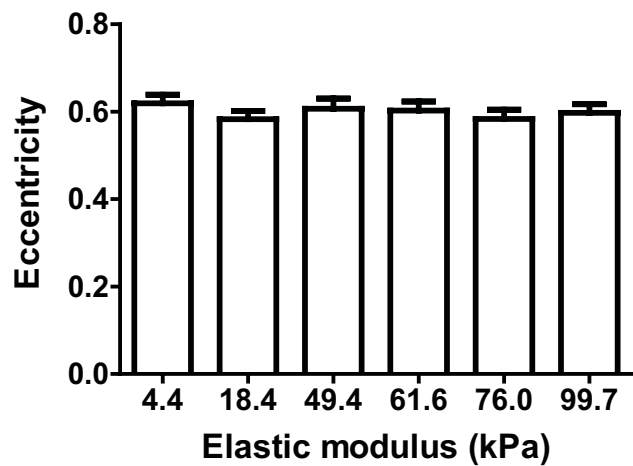

C

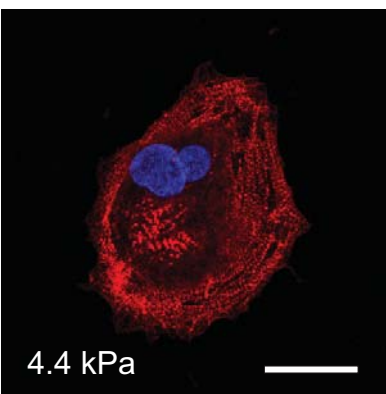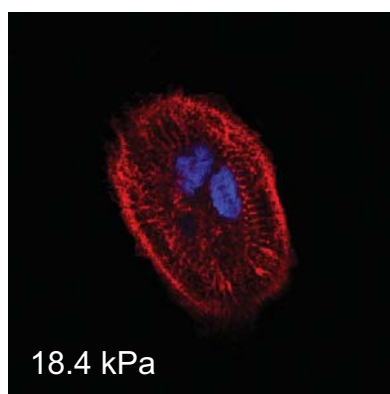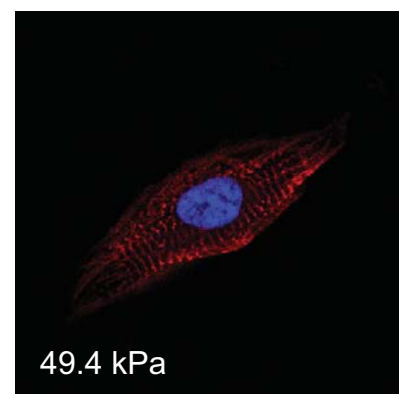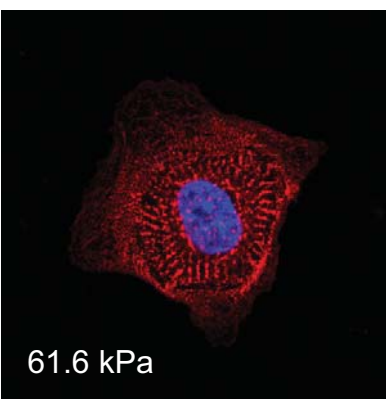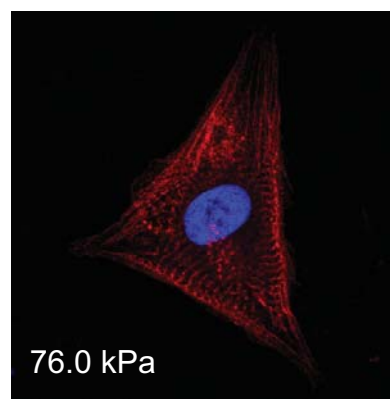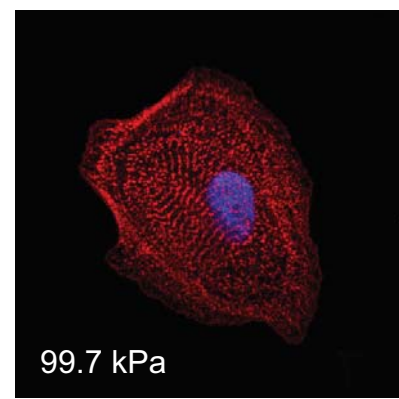

Supplement: Supplementary file 4 [file 508294.f4.pdf]

**A**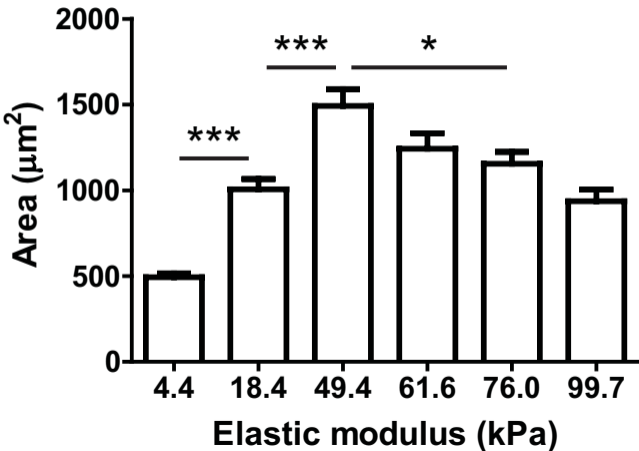**B**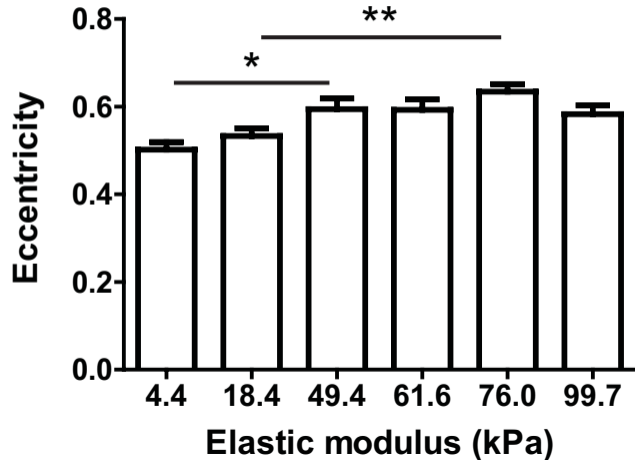

Supplement: Supplementary file 5 [file 508294.f5.pdf]

**A**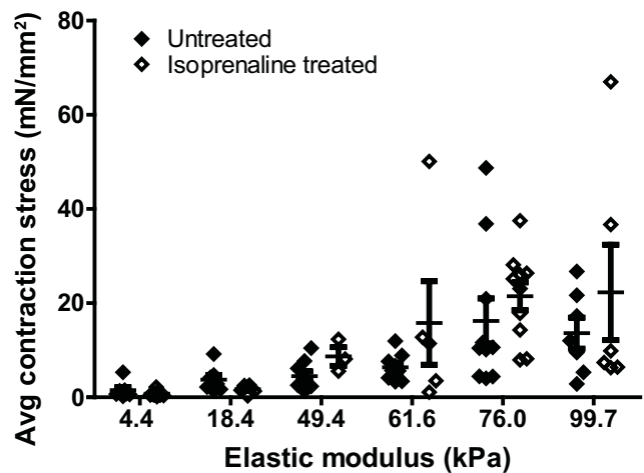**B**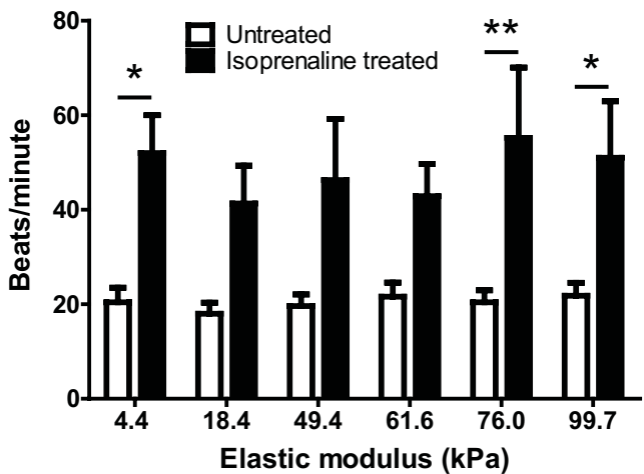

Supplement: Supplementary file 6 [file 508294.f6.pdf]

**A**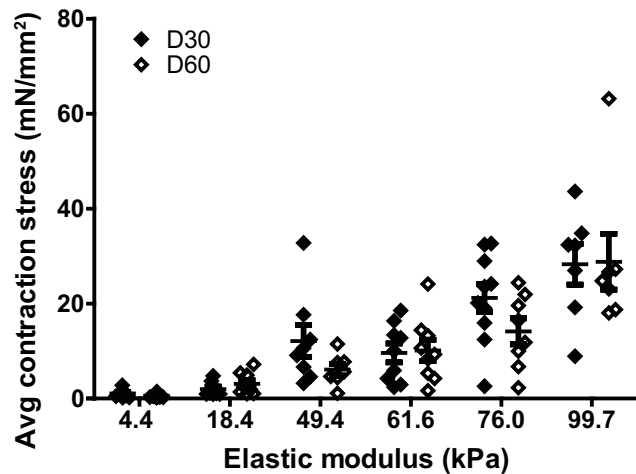**B**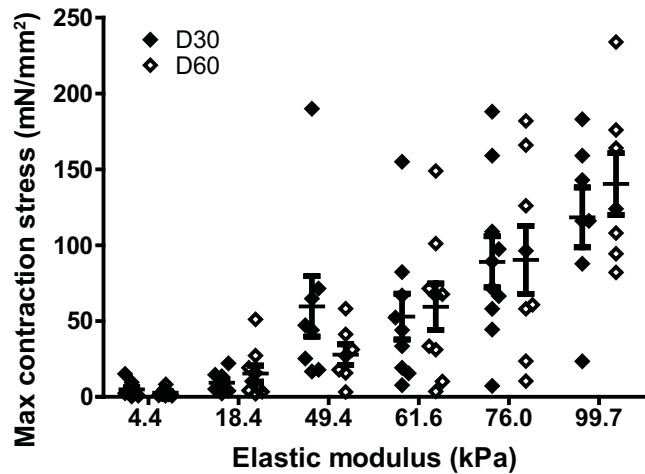**C**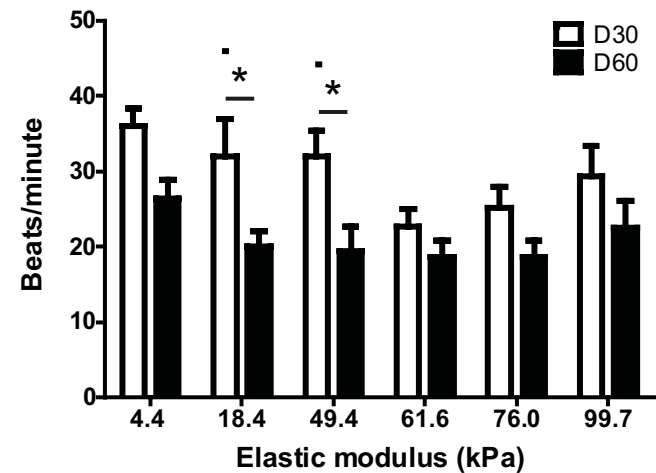

Supplement: Supplementary file 7 [file 508294.f7.pdf]
