# Supplementary material for: Effects of Substrate Mechanics on Contractility of Cardiomyocytes Generated from Human Pluripotent Stem Cells
Source: Int J Cell Biol. 2012 May 9;2012:508294. doi: 10.1155/2012/508294 (PMC3357596; doi:10.1155/2012/508294)
Supplement: Supplementary file 12 [file 508294.f12.pdf]

**Supplementary Table 1.** Parameter values input to the LIBTRC templet file and used to obtain contraction stress data. For parameters not listed in this table, the default values were used.

| Parameter | Description | Value | Units (if applicable) | Source |
|---|---|---|---|---|
| **YMODU** | Elastic modulus of substrate | 4.400E+03 to 9.970E+04 | Pa | Tensile testing of PA hydrogels (see Figure 1) |
| **POSRA** | Poisson's ratio of substrate | 3.000E-01 | | Assumed value for all PA compositions |
| **TFILM** | Thickness of substrate | 1.000E-03 | m | Thickness of PDMS spacers used to generate PA hydrogels |
| **BDIAM** | Diameter of fluorescent beads | 7.200E-07 | m | Label on fluorescent bead packaging |
| **ZBAVE** | Average depth of beads below surface of surface | -2.000E-06 | m | Z-stack of a typical substrate using Nikon A1R confocal microscope |
| **NPIXX** | X dimension of image | 5.120E+02 | pixels | Image property |
| **NPIXY** | Y dimension of image | 5.120E+02 | pixels | Image property |
| **NBITS** | Number of bits/pixel of image | 1.600E+01 | | Image property |
| **ZDEEP** | Depth of optical section | 3.010E-06 | m | Obtained from Nikon NIS-Elements software |
| **MPPX** | Width of pixel in the X direction | 2.100E-07 to 3.100E-07 | m | Depends on additional zoom of data set |
| **MPPY** | Height of pixel in the Y direction | 2.100E-07 to 3.100E-07 | m | Depends on additional zoom of data set |

Reference: Dembo, M. (2010) The LIBTRC User's Guide for Version 2.4.
